# Prognostic factors affecting survival of patients with cancer-related pericardial effusion managed by surgery

**DOI:** 10.1186/1477-7819-12-249

**Published:** 2014-08-05

**Authors:** Hyun Woo Jeon, Deog Gon Cho, Jae Kil Park, Kwan Yong Hyun, Si Young Choi, Jong Hui Suh, Young-Du Kim

**Affiliations:** 1 Department of Thoracic and Cardiovascular Surgery, Bucheon St. Mary’s Hospital, College of Medicine, The Catholic University of Korea, 222 Banpo-daero, Seoul 137-701, Seocho-gu, Republic of Korea; 2 Department of Thoracic and Cardiovascular Surgery, St. Vincent’s Hospital, College of Medicine, The Catholic University of Korea, 222 Banpo-daero, Seoul 137-701, Seocho-gu, Republic of Korea; 3 Department of Thoracic and Cardiovascular Surgery, Seoul St. Mary’s Hospital, College of Medicine, The Catholic University of Korea, 222 Banpo-daero, Seoul 137-701, Seocho-gu, Republic of Korea; 4 Department of Thoracic and Cardiovascular Surgery, Uijeongbu St. Mary’s Hospital, College of Medicine, The Catholic University of Korea, 222 Banpo-daero, Seoul 137-701, Seocho-gu, Republic of Korea; 5 Department of Thoracic and Cardiovascular Surgery, Incheon St. Mary’s Hospital, College of Medicine, The Catholic University of Korea, 222 Banpo-daero, Seoul 137-701, Seocho-gu, Republic of Korea

**Keywords:** Pericardial effusion, Cancer, Pericardial window

## Abstract

**Background:**

Although pericardial effusion (PE) is not uncommon in patients with cancer, it may lead to cardiac tamponade, a life-threatening condition. Prompt life-saving treatment is essential, and also allows the continuation of the cancer treatment. The aim of this study was to determine the prognostic factors for survival in patients with cancer who were treated surgically for PE.

**Methods:**

We retrospectively reviewed the medical records of 55 patients with cancer with PE between January 2003 and October 2012, who were treated with a pericardial window operation. Overall survival (OS) was estimated from the date of surgery, and patients were followed until the time of the final visit or time of death. Clinical outcomes and candidate prognostic factors were analyzed.

**Results:**

The median age of patients was 57 years (range 29 to 82 years), and 31 patients (56.4%) were male. The most common primary malignancy was lung cancer (65.5%), followed by breast cancer (10.9%). Fifteen patients (27.3%) developed recurrence of PE after surgery. The median OS duration was 4 months (range 0 to 39 months). Multivariate analysis found that evidence of pericardial metastasis on preoperative imaging (*P* = 0.029) and confirmation of malignant cells in the PE and/or pericardial tissue (*P* = 0.034) were associated with reduced OS.

**Conclusion:**

Evidence of pericardial metastasis on preoperative imaging and cytopathologic confirmation that the PE and/or pericardial tissue are positive for malignant cells can be used to predict poor clinical outcomes in patients with cancer-related PE.

## Background

Pericardial effusion (PE) associated with malignancy may lead to cardiac tamponade, a life-threatening condition. Lung cancer is the most common primary malignancy associated with PE, followed by breast cancer and lymphoma [1,2]. Most patients complain of a gradual onset of fatigue and shortness of breath [3]. Because of the gradual onset of symptoms, which might be attributed to the underlying malignancy, the diagnosis of malignant PE can be missed or delayed. Although the survival of patients with malignant PE is known to be very short [4], optimal treatments should be commenced immediately to relieve symptoms, allow the continuation of systemic therapy for the primary malignancy, and prevent unexpectedly early death. Since 1829, when Larrey performed surgical drainage to treat PE through the subxiphoid approach [5], various methods, including thoracotomy, video-assisted thoracic surgery (VATS), and laparoscopic surgery, have been used to treat PE associated with various conditions. However, not all cancer-related PE is malignant PE, so the differential diagnosis of cancer-related PE is difficult [6]. In this study, we investigated patients who were treated surgically for cancer-related PE, in order to identify prognostic factors affecting survival.

## Methods

This retrospective study was approved by the institutional review board of the College of Medicine, (Catholic University of Korea). Between January 2003 and October 2012, 139 patients underwent pericardial window surgery for PE associated with various conditions. Patients with and patients without cancer who had PE associated with transudate PE, tuberculosis, bacterial infection, uremia, or autoimmune disease were excluded from this study. Finally, we reviewed the medical records of 55 patients with clinically malignant PE who had undergone surgical management because of cancer-related PE.

**Figure 1:**
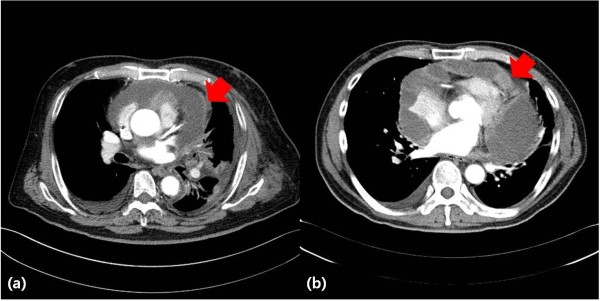
**Computed tomography finding suspicious of pericardial metastasis. (a)** Pericardial wall thickening (arrow) and **(b)** pericardial nodularity (arrow).

Preoperative assessments included chest computed tomography (CT) and two-dimensional (2-D) and Doppler echocardiography. The definition of cardiac tamponade was based on the following criteria [7]: right atrial and ventricular collapse and greater than 25% respiratory variation in mitral inflow. Pericardial metastasis was defined as pericardial nodules, pericardial thickening, or diffuse enhancement of the pericardium on preoperative CT after contrast injection (Figure 1) [8]. The demographic and clinical data of patients and cytopathologic and histopathologic data from the surgical specimens were collected for analysis.

### Statistical analysis

All statistical analyses were carried out using SPSS software (v18l IBM Corporation). Continuous variables were compared using the Kruskal-Wallis test, and categorical variables were compared using the χ^2^ test. Overall survival (OS) was analyzed using the Cox proportional hazards model; before application of this model, the proportionality assumption was checked. Multivariate analysis for OS was also performed using the Cox proportional hazards model. Variables with *P* < 0.05 by univariate analysis were ultimately evaluated by multivariate analysis. *P* < 0.05 was considered statistically significant in multivariate analysis.

## Results

The characteristics of the study patients are shown in Table 1. The median age of patients was 57 years (range 29 to 82 years) and 31 patients (56.4%) were male. The primary malignancy was lung cancer in 36 patients; breast cancer in 6; gastrointestinal cancer in 5; hematologic malignancy in 4; and thyroid cancer, ovarian cancer, thymoma, and cardiac sarcoma in 1 patient each. Seven (12.7%) patients were diagnosed with PE at the time of diagnosis of the primary cancer, and the others were diagnosed during chemotherapy for advanced primary cancer. The median time interval between the diagnosis of cancer and PE was 9 months (range 0 to 180 months). Fifteen patients underwent pericardiocentesis before operation, and of these, eight developed recurrent PE. Evidence of pericardial metastasis on the preoperative CT scan was seen in 20 patients (36.4%). The sensitivity and specificity of CT for the diagnosis of malignant PE (compared with cytopathologic analysis of PE or pericardial tissue) was 35.3% and 61.9%, respectively.

**Table 1:** **Characteristics of 55 patients with pericardial effusion treated by surgery**^**a**^

**Variables**	**Value**
Age, years; mean (range)	57 (29 to 82)
Gender, n (%)	
Male	31 (56.4)
Female	24 (23.6)
Malignancy, n (%)	
Lung cancer	36 (65.5)
Hematologic malignancy	4
GI cancer	5
Breast cancer	6
Ovary cancer	1
Thyroid cancer	1
Cardiac sarcoma	1
Thymoma	1
Time to PE after cancer diagnosis. months	9 (0 to 180)
Dyspnea, n (%)	45 (81.8)
Electrocardiography, n (%)	
Normal	23 (41.8)
Abnormal	32 (58.2)
Sinus tachycardia	20
Low voltage	9
Atrial fibrillation	2
APC	1
Cardiac tamponade, n (%)	28 (50.9)
Ejection fraction, (%)	62% (48 to 75)
Prior pericardiocentesis, n (%)	15 (27.3)
Recurrence after pericardiocentesis, n	8
Maximum distance of pericardial space by CT, mm	32.25 (11.7 to 54.68)
Concomitant pleural effusion, n (%)	39 (70.9)
Mediastinal lymphadenopathy, n (%)	40 (72.7)
Prior radiotherapy of the chest, n (%)	22 (40)
Pericardial metastasis by CT, n (%)	20 (36.4)
Pulmonary thromboembolism, n (%)	3 (5.5)
Extrathoracic metastasis, n (%)	32 (58.2)

*APC*, Atrial premature contraction; *CT*, Computed tomography; *GI*, Gastrointestinal; *PE*, Pericardial effusion.

^a^Data are presented as median (range) unless otherwise stated.

Three different approaches were used for pericardial window surgery: mini-thoracotomy for 14 patients (25.5%), the subxiphoid approach for 16 (29.1%), and VATS for 25 (45.5%. The median anesthesia time was 80 minutes (range 30 to 180 minutes), and the median amount of drainage was 500 ml (range 100 to 1500). Cytopathologic examination of pericardial fluid and pericardial tissue confirmed malignancy in 34 patients (61.8%).

There were two postoperative deaths (3.6%). One patient developed active bleeding after pericardiocentesis, and despite emergent pericardial window surgery to control the bleeding, he died of acute renal failure and hypoxic brain damage on postoperative day 5. The second patient developed pneumonia after surgery and died of sepsis.

There were seven (12.7%) patients with operative morbidity, which included atrial fibrillation, prolonged mechanical ventilation, refractory hypotension, and constrictive pericarditis (Table 2).

**Table 2:** **Operative and postoperative data**^**a**^

**Variables**	**Value**
Operative procedure, n (%)	
VATS	25 (46)
Mini-thoracotomy	14 (25)
Subxiphoid approach	16 (29)
Operative time, min	80 (30 to 180)
Volume of drainage fluid, ml	500 (100 to 1500)
Nature of pericardial fluid, n (%)	
Serous	18 (32.7)
Sanguineous	37 (67.3)
Malignant cells on cytopathology n (%)	34 (61.8)
Adjuvant chemotherapy after operation, n (%)	51 (92.7)
Death, n (%)	2 (3.6)
Complications, n (%)	7 (12.7)
Acute renal failure, n	1
Pneumonia, n	1
Atrial fibrillation, n	1
Prolonged ventilation, n	1
Cardiogenic shock, n	1
Constrictive pericarditis, n	2
Recurrence, n (%)	15 (27.3)

*VATS,* video-assisted thoracic surgery.

^a^Data are presented as median (range) unless otherwise stated.

Fifteen patients (27.3%) developed recurrent PE after surgery. Ten patients (66.7%) with pathologically malignant PE and 6 patients (40%) with pericardial metastasis on preoperative CT showed recurrent PE. During the follow-up period, 45 (81.8%) patients died because of progression of their malignancy. The median survival time was 4 months (range 0 to 39 months), and the 1-year survival rate was 21.8%. There were no significant differences in the rate of postoperative complications and recurrence between the different surgical approaches. However, the anesthesia time was significantly longer for the patients undergoing VATS, compared with the other procedures (*P* = 0.046).

**Figure 2:**
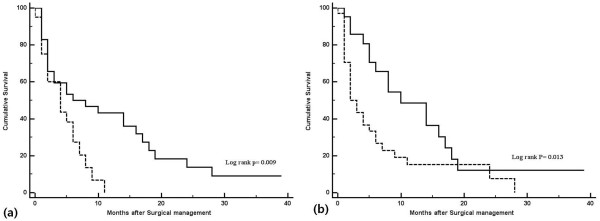
**Overall survival (OS) of patients with cancer-related pericardial effusion. (a)** Overall survival (OS) according to evidence of pericardial metastasis on preoperative imaging. Solid line: evidence of pericardial metastasis (n = 20); dashed line: no evidence of pericardial metastasis (n = 35). **(b)** OS according to cytopathologic confirmation of malignancy. Solid line, confirmation (n = 34); dashed line, no confirmation (n = 21).

**Figure 3:**
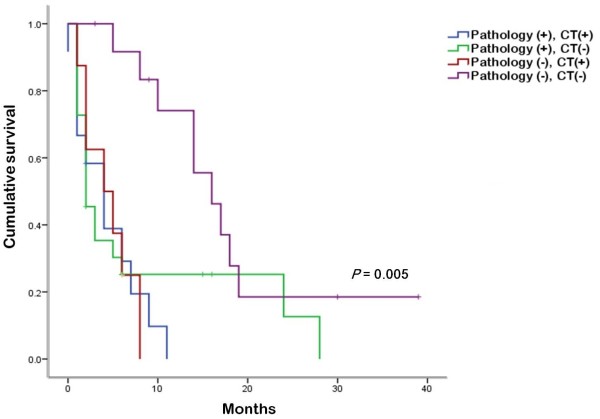
**Overall survival (OS) according to presence of cytopathologically malignant pericardial effusion (PE) or pericardial metastasis by computed tomography (CT).** Patients negative for both pericardial metastasis on CT and cytopathologic malignancy had better clinical outcomes. CT (+), pericardial metastasis on CT scan; pathology (+), cytopathologic malignant PE.

In survival analyses, no affect on OS was seen for age; gender; type of primary malignancy; disease-free interval between the diagnosis of cancer and occurrence of PE; abnormal electrocardiogram; presence of cardiac tamponade; mediastinal lymphadenopathy, pleural effusion, or extrathoracic metastasis; or surgical approach. Evidence of pericardial metastasis on preoperative CT (*P* = 0.029) and malignant PE found on postoperative cytopathologic examination (*P* = 0.034) were associated with poor OS by both univariate and multivariate analysis (Table 3). The median OS times of patients with and without evidence of pericardial metastasis on preoperative CT were 4 and 5 months, respectively. The median OS times of patients with and without cytopathologic confirmation of malignant PE were 2 and 8 months, respectively (Figure 2). Patients negative for both pericardial metastasis on preoperative CT and cytopathologic maliganancy had better clinical outcomes compared with positive for either (Figure 3).

**Table 3:** Univariate and multivariate analysis for overall survival

	**Univariate analysis**			**Multivariate analysis**		
	**HR**	**95% CI**	***P *** **value**	**HR**	**95% CI**	***P *** **value**
Lung cancer	1.153	0.623 to 2.136	0.651	–	–	–
Adenocarcinoma	1.128	0.623 to 2.042	0.691	–	–	–
Interval between diagnoses of PE and primary cancer	0.994	0.981 to 1.007	0.384	–	–	–
Mediastinal lymphadenopathy	1.456	0.735 to 2.884	0.281	–	–	–
Pericardial metastasis on CT	2.224	1.148 to 4.306	0.018	2.078	1.077 to 4.012	0.029
Extrathoracic metastasis	1.818	0.980 to 3.373	0.058	–	–	–
Abnormal EKG	1.730	0.935 to 3.201	0.081	–	–	–
Cytopathologic malignant PE	2.079	1.114 to 3.878	0.021	1.964	1.053 to 3.663	0.034

*PE*, pericardial effusion, *CT*, computed tomography.

Cytopathologically malignant PE included positive PE and/or positive pericardial tissue.

## Discussion

PE is a not uncommon condition. Its etiologies include uremia, malignancy, infection, and autoimmune disease [9]. The clinical presentation at the time of diagnosis varies, because PE generally develops gradually. However, PE in patients with cancer often has an acute onset and may be life-threatening (that is, cardiac tamponade). The mechanisms of PE associated with malignancy include metastasis to the pericardium, obstruction of lymphatic drainage, and induction by drugs or radiation = d [10]. Several studies [4,11,12] have shown that patients with cancer who developed PE had worse clinical outcomes than other patients with PE, with median survival times ranging from 3.7 to 6 months and 1-year survival rates from 13.8% to 20%. The results are similar to our findings of a median survival time of 4 months and a 1-year survival rate of 21.8%.

Although the prognosis of malignant PE is not good, ongoing treatment is sometimes necessary. The goal of treatment is sufficient drainage of the pericardial fluid to relieve the symptomsand prevention of recurrence. Pericardiocentesis is an easier and less invasive procedure than pericardial window surgery, allowing prompt treatment at the time of diagnosis. However, pericardiocentesis has a recurrence rate of up to 20% at 30 days [13], which is higher than recurrence after surgical drainage (1-10% of recurrence) [4,13,14]. In addition, pericardiocentesis may lead to severe complications, such as severe bleeding and cardiac arrest, as was seen in one of our patients who died of his complications.

Surgical drainage is also used for PE, because it is effective and has a low recurrence rate. Since Larrey performed the subxiphoid approach in 1829 [5], various surgical techniques have been used; however, there is no consensus on the best procedure. Subxiphoid pericardiostomy is very simple and safe because it can be performed under direct visualization and under local anesthesia [9]. The disadvantage of this procedure is that pericardial resection is too limited to provide biopsy tissue. Some surgeons prefer the transthoracic approach to the subxiphoid, because it provides better exposure and allows more pericardial resection and natural drainage of effusate to the pleural cavity.

Because of the increased rate of respiratory complications after thoracotomy [15], methods such as mini-thoracotomy and VATS have been introduced. Pericardial window surgery using mini-thoracotomy is a rapid and simple technique [16], and results in less postoperative pain and decreased immune response compared with conventional thoracotomy. Celik *et al*. reported on 48 patients with malignant PE who were treated using mini-thoracotomy [17]. The recurrence rate was only 2.08%, and the 30-day mortality rate was 8.33%. VATS is also minimally invasive surgery, which results in decreased pain and shortened recovery time, and allows more precision because of the magnified field of view [18]. However, VATS require single-lung ventilation, which some patients cannot tolerate. Although comparing the outcomes of the different surgical approaches was difficult because of the heterogeneity of our study patients, there were no significant differences seen for rates of complication or recurrence.

The differential diagnosis between benign and malignant PE is often difficult, and the diagnostic criteria for preoperative imaging of malignant PE have not yet been established. Sun *et al*. [19] suggested that irregular pericardial thickening with PE on CT was highly specific (97.8%) for pericardial metastasis, although the sensitivity (35.7%) was low. We evaluated the diagnostic characteristics of preoperative CT used for pericardial metastasis. Although the sensitivity and specificity of positive CT findings were low (35.3% and 61.9%, respectively), positive preoperative CT findings were associated with shorter survival times (Figure 2a).

Considering the high rate of complications and recurrence in our study, less invasive and well-tolerated procedures may be needed compared with surgical procedures in patients with PE. Ruiz-Garcia *et al*. reported on the use of percutaneous balloon pericardiotomy for the maliganant PE and its effectiveness and safety [20].

There have been several studies to identify the prognostic factors of cancer-related PE. One study found that the sensitivity of pericardial fluid analysis/pericardial biopsy for malignant PE was relatively low compared with that of pericardoscopy (75% and 65%, respectively) and negative results for malignancy of the pericardial fluid and tissue does not mean that PE is non-malignant in patients with cancer [21], so the prognostic roles of pericardial fluid analysis and biopsy are controversial. Wang *et al*. [6] reported that in 60 of 88 patients (68%) with PE-associated non-small cell lung cancer, the pericardial fluid cytology was positive for malignant cells; however, this was not significantly associated with OS. Cullinane *et al*. [3] reported similar results: of 63 patients with cancer, 28 (44%) and 15 (24%) were diagnosed with malignant PE by pericardial fluid cytology and pericardial biopsy, respectively. There was no significant difference in OS between the patients diagnosed by fluid cytology and those diagnosed by biopsy tissue. By contrast, Celik *et al*. [17] reported that in a group of patients with cancer, those with malignant PE had worse clinical outcomes than those with non-malignant PE. Of 48 patients with cancer, 26 (54.1%) developed malignant PE. The mean survival time of patients with and without malignant PE was estimated to be 11.9 and 18.4 months, respectively (*P* = 0.004). Gornik *et al*. investigated 269 patients who had undergone pericardiocentesis for PE, and found that 96 patients had PE-associated malignancy. For these patients, median survival times were 7.3 and 19.7 weeks for patients with and without abnormal cytology, respectively (*P* = 0.0221) [22]. In our study, we found that presence of pericardial metastasis on preoperative CT scans and cytopathologic confirmation of malignant PE were both associated with worse clinical outcomes in multivariate analysis. Furthermore, the patients with cancer without pericardial metastasis on preoperative CT and cytopathologic malignancy had prolonged survival. Although the differential diagnosis of cancer-related PE is difficult, it could be possible to predict prognosis using preoperative CT and cytopathologic confirmation.

Our study has some limitations. It was retrospective study with a small and heterogeneous sample size.

## Conclusions

Surgical treatment of malignant PE is crucial for symptomatic control and precise diagnosis. In the current study, none of the surgical approaches used was found to be superior to the others, therefore, the type of procedure should be based on the individual patient. In addition, presences f pericardial metastasis on preoperative CT and cytopathologic confirmation of malignant PE may be associated with worse clinical outcome.

## Abbreviations

APC: Atrial premature contraction; CT: Computed tomography; GI: Gastrointestinal; PE: Pericardial effusion; VATS: Video-assisted thoracic surgery.

## Competing interests

The authors declare that they have no competing interests.

## Authors’ contributions

HWJ carried out the review of medical records and analysis, and wrote the paper; DGC and SYC reviewed the medical records and revised the paper; JKP and KYH reviewed the medical records; JHS was carried out revision of the analysis; and Y-DK carried out revision and analysis and is the corresponding author. All authors read and approved the final manuscript.
